# Molecular detection of Enteropathogens from diarrheic stool of HIV positive patients in Gondar, Ethiopia

**DOI:** 10.1186/s12879-018-3265-8

**Published:** 2018-07-31

**Authors:** Lubaba Seid, William Stokes, Abebe Genetu Bayih, Sisay Getie, Aberham Abere, Habtie Tesfa, Dylan R. Pillai

**Affiliations:** 1 Department of Medical Parasitology; School of Biomedical and Laboratory Sciences, College of Medicine and Health Sciences, University of Gondar, Gondar, Ethiopia; 2 Department of Medicine, Cumming School of Medicine, University of Calgary, Calgary, Canada; 3 Department of Pathology and Laboratory Medicine, Cumming School of Medicine, University of Calgary, Calgary, Canada; 4 Departments of Pathology, Medicine, and MIID, University of Calgary, 9-3535 Research Road NW, Calgary, AB 1W-416 T2L2K8 Canada

**Keywords:** HIV, Enteropathogens, Molecular, Diarrhea, Diagnostics

## Abstract

**Background:**

Infectious diarrhea is a common problem in the developing world, especially among people living with HIV/AIDS. Traditional diagnostic methods such as stool culture and microscopic examination are limited by resources and poor sensitivity. The use of molecular diagnostics for enteropathogen detection in this region of sub-Saharan Africa has not been fully explored. We sought to identify risk factors and characterize enteropathogens from diarrheic stools of HIV-positive patients in Gondar, Ethiopia using multiplex molecular panels targeting key infectious agents.

**Methods:**

A cross-sectional study of 100 stool samples was performed. Samples were collected consecutively from HIV- positive patients presenting with diarrhea at University of Gondar Hospital clinic, a major center in NW Ethiopia. Genomic DNA was extracted from stool and processed using a multiplex molecular panel Allplex™ [Seegene, Canada]. Correlations between patient characteristics, symptoms, public health risk factors, and enteropathogen type (s) were studied. Eighty-six samples were successfully analyzed by molecular methods.

**Results:**

The mean age was 35 with 43% male. Eighty percent lived in an urban area, 18% had access to well water only, and 81% practiced proper hand hygiene. The majority of patients (72%) were receiving HAART with a median CD4 cell count of 362/μL. Multiple pathogens were detected in 94% of specimens, with an average of 5 enteropathogens per sample. Common bacteria, viruses, and parasites detected were *Shigella spp./*enteroinvasive *E. coli* (80%), enterotoxigenic *E. coli* (73%), *Norovirus* (16%) and *B. hominis* (62%). CD4 cell count < 500/ μL was associated with the presence of viruses (*p* = 0.004) and the absence of STEC (*p* = 0.010). The use of HAART or CD4 levels was not associated with the number of enteropathogens detected.

**Conclusions:**

Diarrheic stool from HIV-positive outpatients in Gondar, Ethiopia had on average 5 enteropathogens present in their stool. *Shigella*spp./*enteroinvasive E. coli* and *enterotoxigenic E. coli* are the major pathogens, not dissimilar to immunocompetent individuals in low income countries.

## Background

Diarrheal illness contributes to significant mortality and morbidity within the HIV-positive population [1]. It accounts for 1 in 9 deaths for children worldwide and the death rate increases 11 -fold in children with HIV [2]. Furthermore, diarrhea is associated with reduced quality of life and can cause psychological and social burden on afflicted patients [3, 4]. This is most notable in low-resource settings such as Ethiopia in which HIV/AIDS occurs in 1.5% of adults [5].

The variety of organisms known to cause diarrhea presents an inherent challenge in treatment to the clinician, and, in diagnosis, to the laboratory due to shortage of reagents, laboratory set up and skilled manpower in developing countries. Clinical laboratories currently utilize an array of different methodologies to test for bacterial, parasitic, and viral causes of diarrhea among HIV/AIDS patients, a strategy that suffers from poor sensitivity, potentially long turnaround times, and complicated ordering practices and workflows [6–14]. Additionally, there are limited or no testing methods routinely available for most diarrheagenic *Escherichia coli* strains [15] and certain enteric viruses.

Molecular techniques for enteropathogen detection provides a comprehensive, rapid, and streamlined alternative to conventional methods for the microbiological diagnosis of diarrhea in the laboratory setting. The potential advantages include improved performance parameters, a more extensive menu of pathogens, and a turnaround time as short as 1 h [15]. Diagnostic yields in terms of pathogens detected are also approximately 30% higher and multiple enteropathogens are more commonly detected per sample, occurring in up to 35% of positive samples [16].

Prevalence of enteropathogens is regionally dependent, with stark differences between developed and developing countries. A study conducted in the United States on 1556 diarrheal stools using the Biofire FilmArray® platform detected a wide variety of enteropathogens in approximately half (832) of the stool samples collected. In the study, EPEC (41.8%), *C. difficile* (24.5%), EAEC (13.1%) and norovirus GI/GII (8.4%) were the most prevalent organisms detected in positive samples and 73% had multiple enteropathogens [15]. In contrast, a study on 91 diarrheal stool of symptomatic Peruvians demonstrated higher prevalence of diarrheagenic *E. coli* strains and mixed enteropathogens. The most common organisms detected in their study was *Shigella spp*./enteroinvasive *E. coli* (EIEC) at 93%, *G. lamblia* (70%), enteroaggregative *E. coli* (EAEC) (60%), and enterotoxigenic *E. coli* (ETEC) (48%). In addition, 100% of their samples contained mixed enteropathogens [17]. Our study’s aim was to identify epidemiological risk factors and characterize enteropathogens from diarrheic stools of HIV-positive patients in Gondar, Ethiopia using multiplex molecular panels targeting key infectious agents.

## Methods

### Patient population

Diarrheic stool was collected consecutively from all pediatric and adult patients with HIV who attended an antiretroviral therapy (ART) clinic at University of Gondar Hospital, Ethiopia from January 2016, to May 2016. All patients who had diarrhea, defined as 3 or more loose or watery stools during a 24-h period, were included in the study. Acute and chronic diarrhea was defined as diarrhea lasting less than 2 weeks and greater than 4 weeks, respectively. Patients who were on antibiotics or anti-parasitic agents were excluded. Socio-demographic data was collected via patient questionnaires (created in Amharic) and face-to-face patient interviews. The study protocol was reviewed and approved by research ethics committee of School of Biomedical and Laboratory Sciences, College of Medicine and Health Sciences, University of Gondar (SBMLS/641/08). A permission letter was obtained from ART clinic, University of Gondar Hospital (para/1002/08). Informed assent and written consent was obtained from each study participant after explaining the objective, benefit and risk of the study with vernacular language that the study participant could understand.

### Specimen handling

Diarrheic stool was collected on site using sterile containers. Wet mount microscopy, using direct saline and iodine smear, and modified acid-fast staining was performed for the detection of *Cryptosporidium*, *Isospora* and *Cyclospora* species. Stool microscopy was performed by two experienced laboratory technologists and discordant results were resolved by a local microbiology expert. After microscopy was performed, specimens were stored at 4 °C for 1 month until further molecular testing was performed.

### Molecular testing

Total nucleic acid was extracted from the stool specimens using QIAamp DNA Stool Mini Kit (QIAGEN, Germany) according to the manufacturer’s instructions [18] and subsequently stored at -20 °C. The extracted stool DNA was then shipped to the University of Calgary, in Calgary, Alberta, for molecular analysis using the Allplex™ [Seegene, Canada] kit on the CFX96^TM^ Real-time PCR instrument (Bio-Rad, Canada). The Allplex™ gastrointestinal panel comprises bacteria including *Campylobacter*, *Clostridium difficle* toxin A/B, *Plesiomonas shigelloides*, *Salmonella*, *Vibrio*, *Yersinia enterocolitica*, and diarrheagenic *E. coli/shigella* including Enteroaggregative *E. coli* (EAEC), Enteropathogenic *E. coli* (EPEC), Enterotoxigenic *E. coli* (ETEC), Shiga-like toxin-producing *E. coli* (STEC), *E. coli* 0157 and Shigella/Enteroinvasive *E. coli* (EIEC). Parasites detected included *Cryptosporidum*, *Cyclospora cayetnensis*, *Entamoeba histolytica*, *Giardia lamblia*, *Blastocystis hominis* (BH) and *Dientamoeba fragilis* (DF). Viruses detected included Adenovirus F 40/41, Astrovirus, Norovirus GI/GII, Rotavirus A and Sapovirus. Negative and positive controls were included in all extraction and amplification procedures. Gene targets are considered proprietary at this time (Seegene, Canada).

### Statistical analysis

Correlations between patient characteristics, symptoms, epidemiological risk factors and enteropathogen (s) were explored using STATA (Version 14.1). Categorical variables were analyzed using Pearson chi-squared or Fisher’s exact test. Continuous variables were analyzed using two paired t-test for normally distributed variables, two-sample Wilcoxon rank-sum test for non-normally distributed variables and linear regression or Spearman rank test for two continuous variables.

## Results

Diarrheic stool from 100 patients was collected. Fourteen samples were excluded from the molecular testing arm due to insufficient sample volume or sample processing deficiency. Patient demographics are outlined in Table 1. The mean age was 34.9 with 5 patients under the age of 14. 43.0% of patients were male, 80.2% lived in an urban area, 17.4% had access to well water only, 88.4% had access to proper latrines, and 81.4% practiced proper hand hygiene. Patients median duration of diarrhea was 10 days with 53.5% having acute diarrhea and 7.0% having chronic diarrhea. Median bowel movement frequency per day was 5. The median CD4 cell count was 361.5/μL with 36.1% having CD4 cell counts < 200/μL. The majority (72.1%) of patients were receiving highly active antiretroviral therapy (HAART) at the time of collection.

**Table 1:** Demographics of Included Study Participants (N = 86)

Age, mean (SD)	34.9 (12.2)
Age, median	35
Male (%)	43.0
Urban (%)	80.2
Tap water (%)	82.6
Access to toilet (%)	88.4
Practices proper hand hygiene (%)	81.4
Receiving HAART (%)	72.1
CD4 count/μl, mean (SD)	359.9 (278.2)
CD4 count/μl, median	361.5
CD4 count < 50/μl (%)	12.8
CD4 count 50–200/μl (%)	23.3
CD4 count 200–500/μl (%)	30.2
CD4 count > 500/μl (%)	34.9
Duration diarrhea, median (days)	10
Diarrhea duration < 14 days (%)	53.5
Diarrhea duration 14–30 days (%)	39.5
Diarrhea duration > 30 days (%)	7.0
Frequency diarrhea per day, median	5
Total organisms, mean (SD)	5.2 (2.3)

Out of the 100 specimens, 28 were positive for intestinal parasites using wet mounts and modified acid fast staining. *C. parvum* was the most common parasite at 28.6% followed by *E. histolytica* (17.9%), *G. lamblia* at (14.3%), *C. cayetanensis* (14.3%), *S. stercoralis*, (7.1%) and hookworm (3.6%).

**Figure 1:**
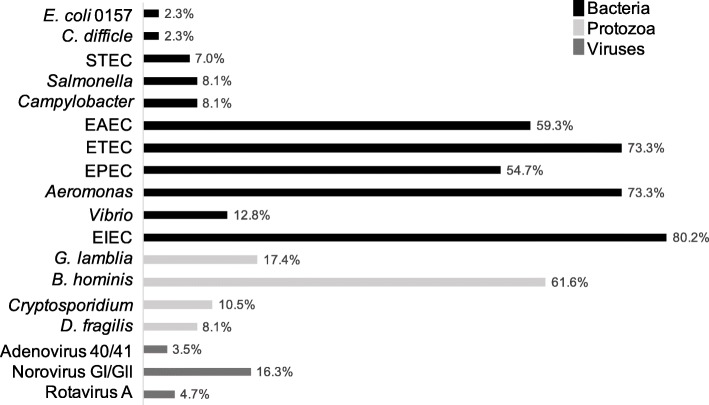
Enteropathogens Detected (%) in HIV Patient Participants with Diarrhea (*N* = 86) using the Allplex™ gastrointestinal panels [Seegene, Canada]

The enteropathogens detected by the Allplex™ panel are outlined in Fig. 1. No failures were detected among the negative and positive controls. Only one patient had no organisms detected (when excluding *B. hominis*, *D. fragilis* and *Aeromonas spp*). The most common organisms detected were *Shigella* spp./enteroinvasive *E. coli* (EIEC) at 80.2%, enterotoxigenic *E. coli* (ETEC) at 73.3%, *Aeromonas spp.* at 73.3%, and enteroaggregative *E. coli* (EAEC) at 59.3%. Parasites detected included *Blastocystis hominis* (61.6%), *Giardia lamblia* (17.4%), *Cryptosporidium spp*. (10.5%), and *Dientamoeba fragilis* (8.1%). Viruses detected included norovirus GI/GII (16.3%), rotavirus A (4.7%) and adenovirus 40/41 (3.5%). Multiple pathogens were detected in 94.1% of stool specimens, with 64.0% having 5 or more enteropathogens (33.7% if excluding *B. hominis*, *D. fragilis* and *Aeromonas spp.*).

Older age was associated with *Campylobacter spp.*, only occurring in patients between over the age of 35 (*p* = 0.04). *D. fragilis* was associated with increased frequency of diarrhea (median 4 movements when *D. fragilis* present compared to median 5, *p* = 0.02). Use of well water was associated with *Cryptosporidium* (33.3% with well water vs 6.0%, *p* = 0.001).

**Fig. 2:**
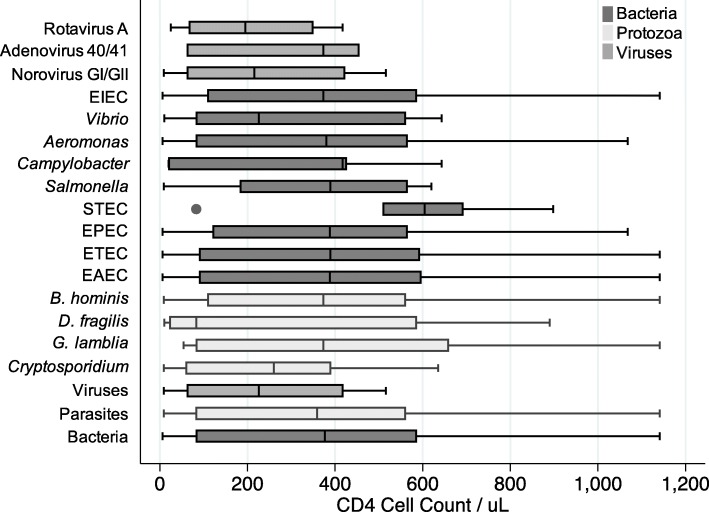
Box Plot graphic (median and interquartile range) depicting CD4 cell counts per μl in relation to the enteropathogen detected (N = 86). *E. coli* 0157 and *C. difficle* excluded from box plot due to insufficient positive cases. The black solid circle denotes a single outlier case

A box plot describing enteropathogen frequencies with CD4 cell count is outlined in Fig. 2. Only viral enteropathogens and STEC were associated with CD4 cell counts. A CD4 cell count < 500/μL was associated with the presence of a viral enteropathogen (*p* = 0.004) and the absence of STEC (*p* = 0.018) (Table 2). Lower CD4 cell counts were also associated with a longer duration of diarrhea (*p* = 0.0015) and older age (*p* = 0.011). Associations between organisms at CD4 count < 200/uL and < 50/uL were analyzed and none were found to be statistically significant.

**Table 2:** Proportion of pathogens detected in patients with CD4 cell counts < 500/μL

Pathogen	CD4 < 500/μL (*N* = 56)	CD4 > 500/μL (*N* = 30)	*p*-value^a^
STEC (%)	1.8	16.7	0.018
Norovirus GI/GII (%)	23.2	3.3	0.029
Rotavirus A (%)	7.1	0	0.293
Adenovirus 40/41 (%)	5.4	0	0.549
Viruses (%)	28.6	3.3	0.004

^a^2-sided Fisher’s exact test

The number of organisms detected in one sample was not associated with any specific demographic, including CD4 cell count, administration of HAART, age, duration of diarrhea or frequency of diarrhea. This was also the case when excluding *B. hominis, D. fragilis* and *Aeromonas spp.*

## Discussion

Detection of intestinal parasites was overall higher using multiplex PCR compared to microscopy in our study. While *Cryptosporidium spp.* detection was similar, multiplex PCR yielded more *G. lamblia*, *B. hominis*, and *D. fragilis* but less *E. histolytica* and *C. cayetanensis*. Notably, microscopy is unable to distinguish *E. histolytica* (pathogen) from *E. dispar* (non-pathogen) whereas molecular detection is specific for *E. histolytica*. Multiplex PCR is known to be more sensitive than conventional techniques for parasite detection therefore it is not clear why *C. cayetanensis* was not detected using multiplex PCR in our study [15]. *Shigella* spp./enteroinvasive enteroinvasive *E. coli* was the most commonly detected organism (s) in our study at 80.2%, followed by ETEC (73.3%), *Aeromonas* spp. (73.3%), EAEC (59.3%) and EPEC (54.7%). This is similar to a study among symptomatic Peruvians in which *Shigella* spp./enteroinvasive *E. coli* was the most commonly detected organism at 93% followed by *G. lamblia* (70%), EAEC (60%), ETEC (48%) and EPEC (41%). *G.lamblia* was of lower prevalence in our study.

In contrast, detection of diarrheagenic *E. coli* strains are much lower when examining stool from patients in developed countries, highlighting their common frequency in sanitation poor settings [19]. Even in asymptomatic individuals, prevalence of diarrheagenic strains of *E. coli* are high in developing countries. For instance, one study detected an enteropathogen in 61% of asymptomatic travelers, with EPEC, EAEC and ETEC being most commonly detected at 42, 28, and 9%, respectively [20].

Our study reported a 24.5% prevalence of enteric viruses with the most common being norovirus GI/GII at 16.3%. This finding is higher than that reported in other studies examining diarrheal stool from adults with HIV/AIDS, which showed an enteric virus prevalence of 15.9–17%. However, those studies were done using less sensitive conventional techniques [21, 22]. A study examining microbiological etiologies of diarrhea in adult Peruvians detected enteric viruses, with multiplex PCR, in 18% of samples, the majority being norovirus GI/GII at 12% [17]. To our knowledge, no study has yet examined diarrheic stool on HIV/AIDS patients using multiplex PCR for comparison.

Debate exists in the literature as to whether *B. hominis*, *D. fragilis* and *Aeromonas spp*. are true enteropathogens with virulence potential [23, 24]. Among several studies examining HIV populations, there has been no statistically significant difference between *B. hominis* found in HIV patients with or without diarrhea [25] and in *D. fragilis* found in HIV-negative or HIV-positive individuals [26]. Several studies on HIV-positive patients did report higher detection of *Aeromonas spp.* in those with diarrhea compared to those without [27, 28]. The lack of a control group, unfortunately, makes our study unable to draw any conclusions with respect to the aforementioned organisms and their intrinsic virulence.

Corresponding to known literature, lower CD4 cell counts in our study were associated with a longer duration of diarrhea [29]. Lower CD4 cell counts were not associated with known opportunistic enteropathogens (*Cryptosporidium* and *Isospora*) but our sample size was small (*N* = 9). We detected an association between lower CD4 cell counts and the presence of enteric viruses (norovirus GI/II, rotavirus A, adenovirus 40/41) in this study. Only one virus was detected when CD4 cell count was > 500/μL. Several studies have associated enteric viruses with more advanced HIV infection, suggesting that CD4 deficiency helps mediate enteric viral replication and infection [22, 30, 31]. Astrovirus has been implicated as a potential opportunistic pathogen but none was detected in this study [32]. A study examining stool from symptomatic HIV patients with diarrhea in London, UK found that adenovirus and rotavirus were associated with significantly lower CD4 cell counts compared to coronavirus or norovirus [21]. Our study found that rotavirus and norovirus were independently associated with lower CD4 cell counts. Adenovirus occurred more frequently at lower CD4 cell counts but was not statistically significant, likely due to small sample size in our study (*N* = 5).

Ninety-four percent of specimens in our study contained a mixture of enteropathogens, with an average of 5 organisms per sample. When excluding *B. hominis, D. fragilis* and *Aeromonas spp.,* prevalence of mixed infection was still high at 90% with a mean of 4 organisms per sample. High prevalence of mixed enteropathogens has been demonstrated in stool from residents and travelers in developing countries. For instance, 25% and 53% of stool specimens from asymptomatic travelers and travelers with diarrhea, respectively, had two or more bacterial pathogens present when using molecular techniques [20]. All stool samples from symptomatic Peruvians had mixed enteropathogens present with a mean of 4.4 pathogens per specimen when tested using the FilmArray® panel [17]. In a longitudinal cohort study of 147 infants in Dhaka, Bangladesh, multiple enteropathogens were also observed in both asymptomatic surveillance and diarrheal stools with the average number of organisms being 4.3 and 5.6, respectively [33]. In contrast, no enteropathogens were detected in non-diarrheal stool samples taken from infants within their first year of life in Virginia, US [33]. Our study has several limitations. Notably, there was no control group for comparison and our overall sample size was not large enough to provide any reliable conclusions on less commonly detected enteropathogens (e.g. *Cryptosporidium spp., Campylobacter spp.*).

Conventional approaches, including culture, microscopy, and antigen-based tests have significant limitations such as the limit of detection and the need for labour-intensive procedures. Molecular diagnostics, especially PCR based tests, are rapidly changing research and practice in infectious diseases. A syndromic approach to the diagnosis of diarrheal disease, with its broad range of potential infectious etiologies, may benefit from the use of multiplex molecular formats. However, their applicability due to cost and capital equipment requirements in developing countries is unclear.

In our study, we found a high number of mixed enteropathogens in diarrheal stools taken from symptomatic HIV patients from Ethiopia when using multiplex PCR. To our knowledge, this is the first study to demonstrate this finding in people living with HIV/AIDS in a developing setting using multiplex molecular methods. Most specimens were mixed with diarrheagenic *E. coli* strains which were similar in prevalence to those seen among diarrheal stools in symptomatic Peruvians [17]. This finding highlights the difficulties in determining colonization versus infection when using extremely sensitive diagnostic methods such as multiplex PCR, and the specific etiological role of each organism in an individual patient. The use of quantitative cutoffs for enteropathogen detection using multiplex PCR has been explored but further clinical studies are needed, especially in developing countries, where high rates of colonization with diarrheagenic strains of *E. coli* exists [33, 34]. One concern is that inappropriate antimicrobial use may escalate with this technology without a fuller understanding of infection versus colonization. Nevertheless, simplification of these technologies, reduction in cost, and better clinical understanding will undoubtedly enhance patient management.

## Conclusion

In conclusion, multiplex PCR panels are elaborate far greater complexity of infection in HIV patients than traditional methods in resource-limited settings. Establishing the true etiology of diarrhea and treatment approach remains challenging. CD4 cell counts < 500/μL were associated with increased detection of gastrointestinal viruses; an interesting finding that requires further investigation.

## Abbreviations

AIDS: Acquired immunodeficiency syndrome
ART: Antiretroviral therapy
BH: *Blastocystis hominis*
DF: *Dientamoeba fragilis*
DNA: Deoxyribonucleic acid
EAEC: Enteroaggregative *E. coli*
EIEC: *Shigella spp*./enteroinvasive *E. coli*
EPEC: Enteropathogenic *E. coli*
ETEC: Enterotoxigenic *E. coli*
HIV: Human immunodeficiency virus
PCR: Polymerase chain reaction
STEC: Shiga-like toxin-producing *E. coli*
